# A nomogram based on pretreatment clinical parameters for the prediction of inadequate biochemical response in primary biliary cholangitis

**DOI:** 10.1002/jcla.23501

**Published:** 2020-09-11

**Authors:** Siyuan Tian, Yansheng Liu, Keshuai Sun, Xia Zhou, Shuoyi Ma, Miao Zhang, Xinmin Zhou, Lu Wang, Ying Han

**Affiliations:** ^1^ Institute of Digestive Diseases Xijing Hospital Fourth Military Medical University Xi'an China; ^2^ State Key Laboratory of Cancer Biology Department of Biochemistry and Molecular Biology Fourth Military Medical University Xi'an China

**Keywords:** autoimmune liver disease, biochemical response, nomogram, primary biliary cholangitis, ursodeoxycholic acid

## Abstract

**Background:**

Ursodeoxycholic acid (UDCA) has been widely recommended as the first‐line drug for primary biliary cholangitis (PBC) in the current guidelines. However, its therapeutic effects are poor in nearly one‐third of patients. The early identification and intervention of these patients is crucial for delaying disease progression. Therefore, we explored risk factors for inadequate biochemical response and constructed a nomogram to predict the potential risk.

**Methods:**

We enrolled 356 patients and randomly divided them into training (70%) and validation groups (30%). We defined inadequate biochemical response as the study endpoint. Logistic analysis was used to identify the independent predictors of poor biochemical response. Based on these factors, a predictive nomogram was finally constructed. Then, discrimination and calibration were evaluated by internal validation. Additionally, the association between the model predictions and prognosis was further analyzed.

**Results:**

Female sex, and albumin and bilirubin concentrations were identified as risk factors, and a nomogram was built based on these factors. The areas under the ROC curves of the training and validation groups were 0.809 and 0.791, respectively. Moreover, calibration curves showed that predictions of the nomogram had good concordance with the actual outcomes. The correlation analysis demonstrated that PBC patients with a high probability of a suboptimal biochemical response were more likely to have adverse outcomes.

**Conclusion:**

We constructed a nomogram, which can accurately predict the risk of inadequate biochemical response to UDCA, facilitating the early screening of high‐risk patients with PBC who should be prioritized for additional therapy.

## Graphical abstract

Flowchart of the study design.

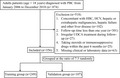

## 1 INTRODUCTION

Primary biliary cholangitis (PBC; formerly referred to as primary biliary cirrhosis) is a chronic autoimmune liver disease characterized by progressive nonsuppurative inflammation, and the destruction of small intrahepatic bile ducts.^1^, ^2^, ^3^ The disease will further deteriorate and even develop into cirrhosis or hepatic failure without early intervention. The survival of some advanced patients is only 3.1 months.^4^ At present, ursodeoxycholic acid (UDCA) has been widely recognized as the most important therapeutic agent with demonstrated improvements in liver biochemistry and liver transplantation‐free survival.^5^, ^6^ In addition, numerous related studies have demonstrated that the efficacy of UDCA strongly determines long‐term outcomes.^4^, ^5^, ^7^, ^8^ The expected survival rate of early PBC patients obtaining good clinical effects was similar to that of the general population.^9^ Similarly, advanced PBC patients responding to UDCA could also benefit from it.^10^ Thus, UDCA is suitable for PBC patients of all stages, especially early‐stage patients.

However, nearly 40% of patients failed to achieve a complete therapeutic response, and their survival rate was unsatisfactory.^9^ Therefore, second‐line agents have been proposed for these patients in the most recent guidelines.^11^ In addition, several different criteria can be used to assess biochemical response to UDCA therapy. However, it takes 1 year or more to evaluate the response. During this period, patients with an active stage have a high risk of disease progression, due to the lack of timely intervention. Therefore, it is very important to reveal the characteristics and related risk factors for high‐risk patients with PBC as early as possible and guide the next step of treatment.

Several studies have attempted to identify factors influencing the inadequate response to UDCA in PBC patients. Nevertheless, different biochemical response criteria were used to evaluate therapeutic efficacy in these studies.^6^, ^12^, ^13^, ^14^ There are significant differences in the criteria for evaluating the UDCA response in PBC patients.^15^ Given the potential risk of misidentification, the comprehensive evaluation of biochemical response can be superior to the use of a single criterion and could be helpful in risk stratification. In this study, we sought to retrospectively collect pretreatment clinical parameters, with the aim of identifying potential nonresponders to UDCA (defined by combined biochemical response criteria), using these clinical parameters to develop and validate a nomogram to facilitate decision‐making. Subsequently, the associations between the UDCA inadequate response model and long‐term outcomes predicted by GLOBE and UK‐PBC scores were analyzed to test the plausibility of the model.

## 2 MATERIALS AND METHODS

### 2.1 Study design and population

This retrospective study was performed on 356 PBC patients diagnosed at Xijing Hospital of the Fourth Military Medical University (Xi'an, China) from January 2006 to December 2018. The patients were randomly divided into the training group (n = 249) and validation group (n = 107) at a ratio of 7:3. All patients meeting the following criteria were eligible for inclusion: (a) age > 18 years, (b) diagnosis of PBC according to the published guidelines (meeting at least two of these conditions: elevated ALP (alkaline phosphatase) levels in serum, positive antimitochondrial antibodies (AMAs) or the AMA‐M2 subtype, liver histology consistent with PBC)^16^; (c) standard UDCA treatment for more than 1 year, 13‐15 mg/kg/d. The exclusion criteria were as follows: (a) concomitant with other liver diseases such as HBV, HCV, hepatic or extrahepatic malignancies, and hepatic failure; (b) follow‐up time less than 1 year; (c) significant loss of data for analysis; and (d) previous treatment with steroids or immunosuppressive drugs within the past 6 months. The study protocol was approved by the Ethics Committee of Xijing Hospital, and informed consent was obtained in writing from each patient.

### 2.2 Clinical and laboratory features of patients

Relevant data were extracted from the electronic medical records and recorded in the database, including sex, age, AMA, hemoglobin, PLT (platelet count), ALT (alanine aminotransferase), AST (aspartate aminotransferase), ALP, GGT (gamma‐glutamyl transferase), ALB (albumin), TBiL (total bilirubin), PT (prothrombin time), and IgM (immunoglobulin M). Among these parameters, ALT, AST, TBiL, ALP, GGT, and ALB were expressed as their respective upper limit of normal (ULN) or lower limit of normal (LLN) to explain interlaboratory variability. Furthermore, three noninvasive indices (AAR,^17^ FIB‐4,^18^ and APRI^19^) reflecting the degree of liver fibrosis were calculated at baseline.

### 2.3 GLOBE score and UK‐PBC risk score

The GLOBE score was used to predict the 5‐, 10‐, and 15‐year probability of liver transplant‐free survival in percentages (%), while the UK‐PBC risk score was used to reflect the risk of liver transplantation or liver‐related death within 5, 10, or 15 years. These scores can provide precise and personalized probability estimates within the defined time points. The related formulas can be obtained from relevant literature.^6^, ^10^

### 2.4 Histological evaluation of liver specimens

Liver biopsies were performed according to standardized procedures. Tissue sections were stained with hematoxylin and eosin and then assessed according to Ludwig's classification. Two pathologists independently evaluated the histological features of PBC. Any differences in opinions were resolved by consensus.

### 2.5 Follow‐up and study endpoints

Once diagnosed with PBC, all patients received standard UDCA therapy as recommended by the guidelines. Each patient had at least one year of follow‐up, during which relevant clinical data were re‐evaluated. The study endpoint was UDCA incomplete response, defined according to the integrated Barcelona criteria^7^ and the Paris Ⅰ criteria.^5^ Specifically, incomplete biochemical response was defined as failure to meet both criteria; otherwise, the patient was considered to have achieved biochemical response.

### 2.6 Statistical analysis

Normally distributed continuous variables are expressed as means ± standard deviations. Nonnormally distributed variables are presented as medians and 25th‐75th interquartile ranges. Student's *t* test and the Wilcoxon signed‐rank test were used to compare two groups, as appropriate. Categorical variables are expressed as rates and were compared using the chi‐square or Fisher's exact test. Correlations were tested using Spearman's rank correlation coefficient. Univariate logistic regression analysis was used to evaluate the relationships between clinical characteristics and inadequate UDCA response. Variables with statistical significance (*P* ≤ .05) in the results were included in the multivariate logistic regression model with a forward stepwise selection process. A nomogram was established based on the results of the multivariate analysis using the “rms” package of R software (version 3.5.1). The “pROC” package was used to plot ROC curves. The area under the curve (AUC) and Hosmer‐Lemeshow test were analyzed to test the discrimination and calibration of the nomogram. Then, the optimum cutoff point was determined by the greatest Youden index, and the sensitivity, specificity, and likelihood ratios were calculated based on the cutoff point. All other statistical analyses were performed using SPSS software version 21.0 (SPSS Inc.) and GraphPad Prism 7 (GraphPad Software Inc.). All *P*‐values were two‐sided, and *P*‐ values ≤.05 were considered statistically significant.

## 3 RESULTS

### 3.1 Demographics and baseline features

A total of 356 patients met the inclusion criteria and were randomly divided into the training group (n = 249) and validation group (n = 107) at a ratio of 7:3. The baseline characteristics, including demographic and clinical features, of the study population are summarized in Table 1. AMA was detected in 275 (77%) of 356 patients with PBC. Stage was available for 251 patients, and there were 168 cases of stages I and II and 83 cases of stages III and IV. The demographics and baseline characteristics were similar between the two groups.

**Table 1:** Baseline characteristics of the training and validation groups

Characteristics	Training group (n = 249)	Validation group (n = 107)	*P‐*value
Sex, female n (%)	206 (82.7)	86 (80.3)	.595
Age (years)	52.5 ± 9.6	53.2 ± 11.3	.933
Positive AMA (%)	202 (83.8)	73 (88)	.365
Hemoglobin (g/dL)	120 (108‐131)	117 (110‐132)	.559
PLT (×10^9^/L)	137 (83‐198)	147 (83‐210)	.598
ALT (×ULN)	1.30 (0.85‐2.09)	1.23 (0.75‐2.01)	.995
AST (×ULN)	1.57 (1.09‐2.28)	1.53 (0.89‐2.84)	.356
ALP (×ULN)	1.77 (1.11‐2.87)	1.78 (0.93‐3.68)	.552
GGT (×ULN)	5.89 (2.38‐9.82)	6.49 (1.65‐10.40)	.620
ALB (×LLN)	0.99 (0.91‐1.05)	0.99 (0.91‐1.05)	.152
TBiL (×ULN)	0.80 (0.56‐1.28)	0.87 (0.58‐1.30)	.719
Prothrombin time (s)	12.9 (12.3‐13.9)	12.9 (12.2‐13.5)	.934
Immunoglobulin M (g/L)	3.01 (1.88‐4.60)	3.29 (2.22‐5.05)	.181
Noninvasive liver fibrosis model			
AAR	1.05 (0.82‐1.36)	1.06 (0.88‐1.44)	.348
FIB‐4	3.02 (1.79‐5.19)	2.94 (1.57‐6.71)	.925
APRI	1.26 (0.76‐2.06)	1.25 (0.65‐2.20)	.971
Histological stage (%)			
Early (I‐II)	124 (66.3%)	44 (18.8%)	.720
Late (III‐IV)	63 (33.7%)	20 (31.2%)	
UDCA response			
Response	198 (79.5%)	83 (77.6%)	.679
Nonresponse	51 (20.5%)	24 (22.4%)	

Note

Continuous variables are shown as mean ± SD or median (IQR) and categorical variables as n (%).

Abbreviations: ALB, albumin; ALP, alkaline phosphatase; ALT, alanine aminotransferase; AMA, antimitochondrial antibody; AST, aspartate aminotransferase; GGT, gamma‐glutamyl transferase; LLN, lower limit of normal; PLT, platelet count; TBiL, total bilirubin; UDCA, ursodeoxycholic acid; ULN, upper limit of normal.

### 3.2 Influencing factors of inadequate biochemical response

The results of univariate analysis with the logistic regression model in the training group are presented in Table 2. According to the results of the univariate analysis, 8 factors were statistically significant, including sex, PLT, ALT, AST, TBiL, GGT, ALB, and APRI. All of these variables were incorporated into the multivariable model. After adjusting for age and sex, the multivariate analysis indicated that TBiL level (odds ratio [OR], 1.973; 95% confidence interval [CI], 1.431‐2.721; *P* < .001) was an independent risk factor, while sex (OR, 0.343; 95% CI, 0.156‐0.753; *P* = .008) and ALB level (OR, 0.019; 95% CI, 0.001‐0.399; *P* = .011) were independent protective factors of inadequate response.

**Table 2:** Univariate and multivariate analyses of influencing factors for inadequate biochemical response in the training group

Variables	Univariate analysis			Multivariate analysis		
	OR	95% CI	*P*‐value	OR	95% CI	*P*‐value
Sex, female n (%)	0.395	0.192‐0.814	.012	0.343	0.156‐0.753	.008
Age (years)	1.019	0.987‐1.051	.255			
Positive AMA (%)	0.823	0.362‐1.871	.642			
Hemoglobin (g/dL)	0.987	0.971‐1.003	.106			
PLT (×10^9^/L)	0.994	0.990‐0.999	.012			
ALT (×ULN)	1.378	1.083‐1.753	.009			
AST (×ULN)	1.383	1.059‐1.804	.017			
ALP (×ULN)	1.167	0.974‐1.399	.094			
GGT (×ULN)	1.063	1.017‐1.112	.007			
ALB (×LLN)	1.014	0.001‐0.250	.004	0.019	0.001‐0.399	.011
TBiL (×ULN)	2.066	1.500‐2.845	<.001	1.973	1.431‐2.721	<.001
Prothrombin time (s)	1.103	0.880‐1.383	.395			
Immunoglobulin M (g/L)	1.042	0.925‐1.174	.496			
Noninvasive liver fibrosis model						
AAR	0.617	0.290‐1.317	.212			
FIB‐4	1.08	0.997‐1.177	.059			
APRI	1.212	1.039‐1.413	.014			

Note

Continuous variables are shown as mean ± SD or median (IQR) and categorical variables as n (%).

Multivariate regression analyses were carried out after adjusting for sex and age.

Abbreviations: ALB, albumin; ALP, alkaline phosphatase; ALT, alanine aminotransferase; AMA, antimitochondrial antibody; AST, aspartate aminotransferase; CI, confidence interval; GGT, gamma‐glutamyl transferase; LLN, lower limit of normal; OR, odds ratio; PLT, platelet count; TBiL, total bilirubin; ULN, upper limit of normal.

### 3.3 Development and validation of a predictive nomogram

**Figure 1:**
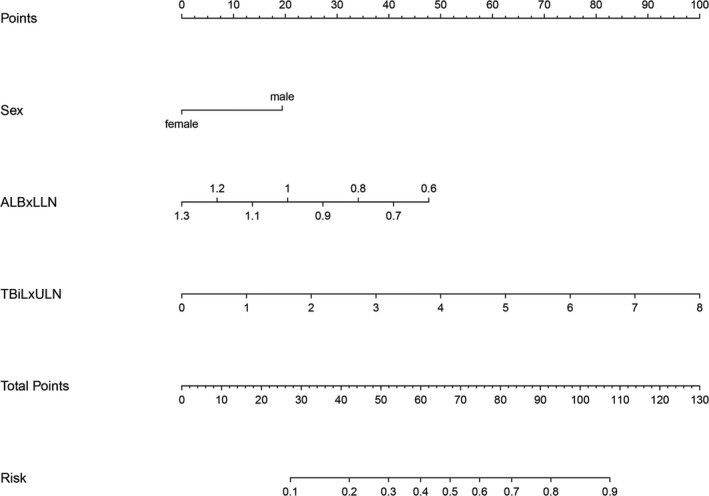
Nomogram to predict the probability of inadequate biochemical response to ursodeoxycholic acid in PBC patients. ALB, albumin; LLN, lower limit of normal; TBiL, total bilirubin; ULN; upper limit of normal

A nomogram incorporating the significant factors in multivariate analysis was established, as shown in Figure 1. The basic method is as follows: Scores corresponding to each factor can be obtained according to the nomogram plot. Next, the sum of the scores assigned to each predictor is the total score of the nomogram, and the corresponding probability is the probability that the patient will have an incomplete UDCA response.

**Figure 2:**
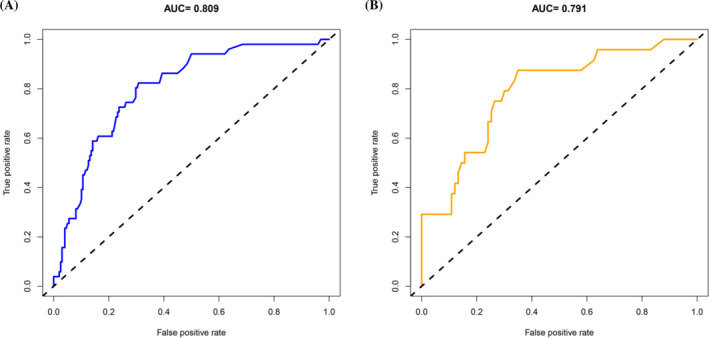
Receiver operating characteristic (ROC) curves for the predictive model. A, ROC curve in the training group. B, ROC curve in the validation group. (AUC, area under the ROC curve)

**Figure 3:**
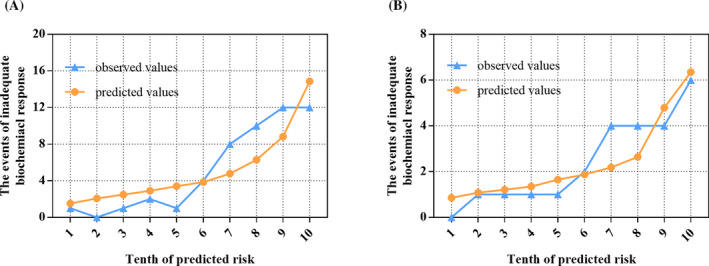
Calibration curves for the predictive model. A, Calibration curve in the training group (χ^2^ = 14.539, *P* > .05). B, Calibration curve in the validation group (χ^2^ = 4.526, *P* > .05)

Then, discrimination and calibration were further analyzed to test the accuracy of the nomogram. The area under the ROC curves of the two groups were 0.809 (95% CI, 0.755‐0.856) and 0.791 (95% CI, 0.690‐0.892) (Figure 2). In the training group, the best cutoff value according to the ROC curves was −1.49, with the highest Youden index at 0.551. At this point, the sensitivity, specificity, positive likelihood ratio, and negative likelihood ratio for the training group were 0.804, 0.747, 3.178, and 0.262, respectively. Applying the cutoff value in the validation group, those corresponding values were 0.750, 0.723, 2.708, and 0.346, respectively. The nomogram showed good discrimination ability in both groups. Next, the Hosmer‐Lemeshow test was used to evaluate the fit of the model in the training group (χ^2^ = 14.539, *P* > .05) and validation group (χ^2^ = 4.526, *P* > .05). Calibration plots of inadequate biochemical response for PBC patients by each tenth of the risk are shown in Figure 3. The plot indicated that the events of inadequate biochemical response were in good agreement with the actual observed results.

### 3.4 Associations between the nomogram predictions and long‐term outcomes estimated by the GLOBE and UK‐PBC score

**Figure 4:**
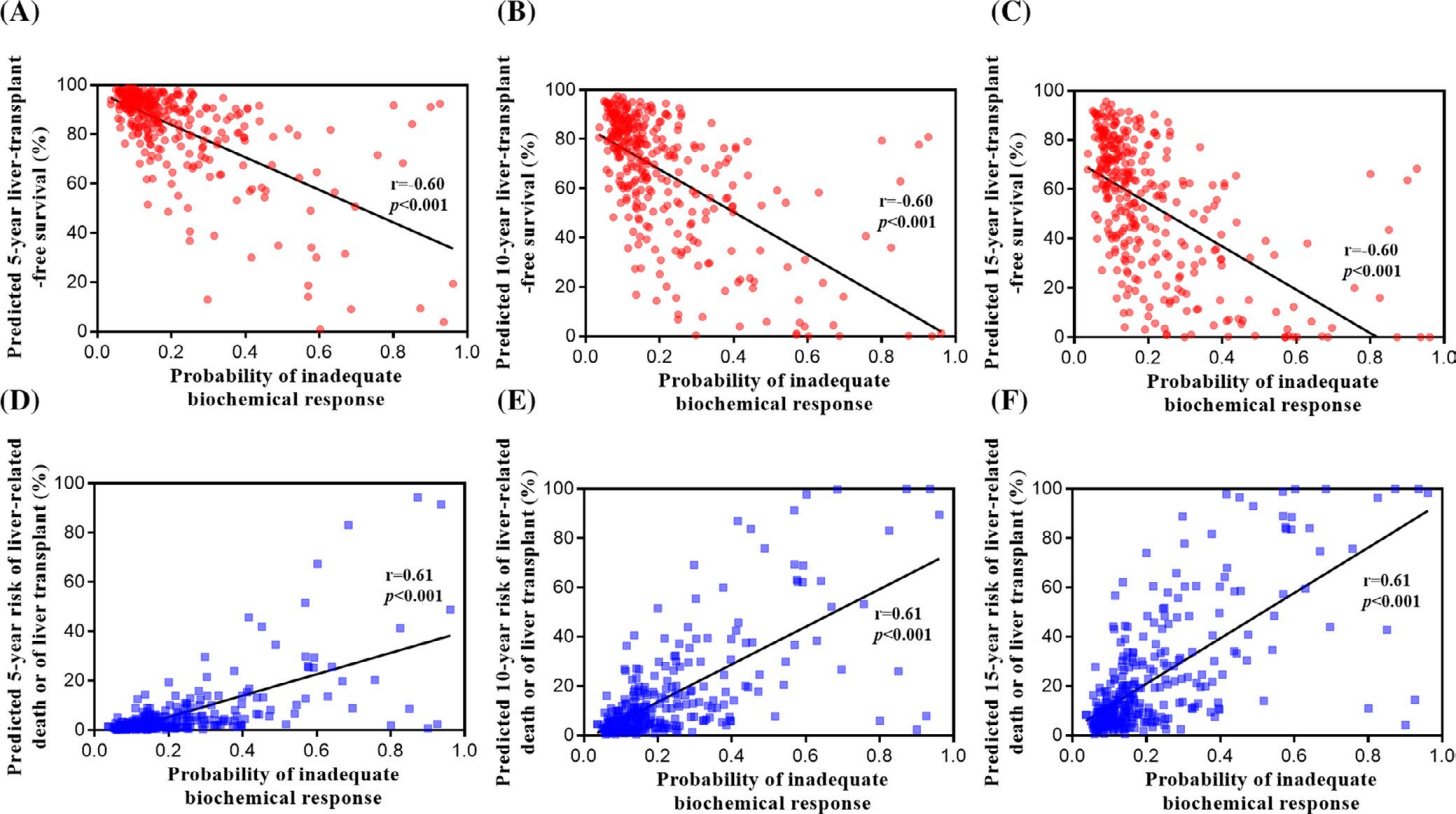
Correlations between the nomogram predictions and long‐term prognosis. A, B, and C, Correlations between the probability of inadequate biochemical response and the predicted liver transplant‐free survival by the GLOBE score. (D, E, and F) Correlations between the probability of inadequate biochemical response and the predicted risk of liver transplant or death by the UK‐PBC risk scores

The UDCA response determines the long‐term prognosis of patients. To verify the reasonability of this model, we correlated the survival status of patients with the predictions of this model. In total, 332 patients were included in the final analysis. The 5‐year, 10‐year, and 15‐year liver transplant‐free survival rates were calculated using the GLOBE score. The predicted liver transplant‐free survival was negatively associated with the probability of an inadequate biochemical response (*r* = −.60, *P* < .001), as illustrated in Figure 4A‐C. This observation indicated that patients with a high risk of incomplete biochemical response displayed a poorer prognosis than those with a low risk. This conclusion was further confirmed by the liver‐related death or liver transplant rates estimated by the UK‐PBC risk score (Figure 4D‐F). As expected, the risk was positively correlated with an increase in the probability of inadequate UDCA response (*r* = .61, *P* < .001).

## 4 DISCUSSION

Although UDCA is widely applied as a basic drug to treat patients with PBC, some patients still have a poor biochemical response after 12 months. The combination of obeticholic acid,^20^ bezafibrate,^21^ and fenofibrate^22^ is expected to improve the therapeutic outcomes and survival of these patients. However, these high‐risk patients appear to wait the longest time for combination therapy. Therefore, it is necessary to develop a tool predicting those who are more likely to have an inadequate biochemical response to UDCA to determine which patients treated with monotherapy need early intervention. In this study, we retrospectively analyzed the potential factors influencing the biochemical response to UDCA. Then, we developed and validated a nomogram for predicting the risk of a suboptimal response to UDCA. This nomogram may serve as a simple tool for clinicians to provide personal medical decision‐making for patients.

Several studies have shown that factors including age, sex, biochemical index, and autoantibodies can influence the biochemical response to UDCA, but no agreement has been reached. Moreover, there are no clear criteria that can reliably predict the response to UDCA.^15^ Therefore, there were limitations to drawing conclusions from a single biochemical response criterion, which may lead to the misclassification of the clinical effect of UDCA in PBC patients. In this study, the Paris I criteria combined with the Barcelona criteria were defined as the study endpoints. Strikingly, following the application of the strict screening criteria, our logistic regression analysis found that only sex, and albumin and bilirubin levels were independent influencing factors of drug response.

Consistent with that in a previous report, in this study, we found that male patients had lower drug response rates than female patients.^13^ Additionally, the results from a single‐center follow‐up study showed that men were more likely to have a shorter survival time than women in PBC patients treated with UDCA.^14^ Among the independent risk factors, the TBiL level at baseline was a strong predictor of inadequate UDCA response, with an OR value of approximately 2. The probability of response declined significantly as the TBiL level increased. Serum bilirubin is not only used for evaluating UDCA response in PBC patients (Paris I, Paris II,^23^ and Rotterdam criteria^9^) but also an important predictor of the GLOBE score^6^ and UK‐PBC risk score.^10^ Furthermore, Corpechot et al^5^ reported that baseline TBiL levels were higher in patients with refractory disease. TBiL ≥17.1 μmol/L before treatment was an independent factor of liver transplantation or death. Albumin is also closely related to liver function. The reduction in serum albumin is currently considered to be an independent predictor of progression to cirrhosis and mortality.^24^ Previous research has indicated that PBC can be classified into three stages according to albumin and serum bilirubin concentrations. They were also considered to be the most consistent prognostic factors with survival.^25^ Our results also suggested that albumin was a protective factor, and its value was positively correlated with biochemical response.

The nomogram built using the above three variables exhibited good predictive value for inadequate biochemical response, with the AUC reaching 0.809. In addition, its calibration also showed good concordance between the predicted and observed results. Furthermore, the correlation between the predictive model and long‐term outcomes predicted using the GLOBE and UK‐PBC scores was analyzed to test the plausibility of the model. In line with our expectations, compared with low‐risk individuals, PBC patients with a high risk of an inadequate UDCA response were more likely to have a poor prognosis. The results further supported the rationality of this model.

Nevertheless, we acknowledge some limitations that deserve comment. First, some patients identified “responders” by the study endpoints may fail to meet the Paris Ⅰ or Barcelona criteria. Nevertheless, there was a need to establish a strict nonresponse criterion to ensure that these patients could be excluded to avoid overtreatment. Our aim was to establish a nomogram that facilitated the early screening of patients who were truly at high risk and need early intervention. Second‐line drug treatment could be applied initially in high‐risk groups without waiting one year for evaluation. By contrast, low‐risk patients could be kept on standard therapy and followed up closely. Second, the validation group and training group were from the same population, which may lead to selection bias. Further large sample validation in different centers is needed. Finally, the relatively short follow‐up period was also a limitation. Whether this nomogram is suitable for predicting hard clinical outcomes, such as death or liver transplantation, needs to be further explored.

In conclusion, we developed a nomogram that could identify potential nonresponders to UDCA at baseline, which could facilitate risk evaluation and stratification for PBC patients.

## AUTHOR CONTRIBUTIONS

YH and LW conceived and designed the experiments. SYT, YSL, KSS, XZ, and SYM performed the experiments. SYT, YSL, and KSS analyzed the data. XZ, MZ, and XMZ collected the samples and interpreted the data. SYT drafted the article. All authors critically reviewed the manuscript.
